# Concordance between five criteria of metabolic syndrome in teenagers from a Peruvian high andes region

**DOI:** 10.17843/rpmesp.2023.402.12546

**Published:** 2023-06-30

**Authors:** Franco Romaní-Romaní, Luis Fernando Pachacama Ramirez, Juan Diego Pichihua Grandez, Diego Maximiliano Guevara Rodríguez, Viviana Cornejo Luyo, Christian Eduardo Sheen Vargas, Juana Aurelia Ninatanta-Ortiz, Martha Vicenta Abanto Villar, Katia Maribel Pérez Cieza, Rosa Ricardina Chávez Farro, Segunda Aydeé García Flores

**Affiliations:** 1 Faculty of Human Medicine, Universidad de Piura, Lima, Peru. Universidad Nacional de Piura Faculty of Human Medicine Universidad de Piura Lima Peru; 2 Academic Professional School of Nursing, Faculty of Health Sciences, Universidad Nacional de Cajamarca, Cajamarca, Perú. Universidad Nacional de Cajamarca Academic Professional School of Nursing Faculty of Health Sciences Universidad Nacional de Cajamarca Cajamarca Peru

**Keywords:** Metabolic Syndrome, Adolescent, Diagnosis, Peru

## Abstract

**Objective.:**

To determine the concordance between five diagnostic criteria for metabolic syndrome (MS) among teenagers from a Peruvian high Andes region.

**Materials and methods.:**

A cross-sectional study was carried out with secondary data from an intervention study in two public schools in 2019. We included 397 teenagers who lived in the city of Cajamarca, in the Andean region of Peru. We applied the criteria from the Adult Treatment Panel III (ATP-III) modified by Cook, the International Diabetes Federation (IDF), the American Heart Association (AHA), Ferranti, and the World Health Organization (WHO). The point prevalence and interval prevalence were estimated with the five criteria. The Kappa concordance coefficient with an 95% confidence interval (95%CI) was estimated.

**Results.:**

The Ferranti criterion identified 17.1% (95%CI: 13.4 to 20.8) of teenagers with MS, followed by the ATP-III criterion with 4.3% (95%CI: 2.3 to 6.3); the other criteria identified a lower frequency. The best concordance was found between the AHA and ATP-III criteria (k = 0.905); the WHO and IDF criteria had a coefficient of 0.628. The five criteria coincided in classifying six adolescents (1.5%) as MS.

**Conclusions.:**

The AHA and ATP-III criteria modified by Cook had almost perfect concordance, which was also found for both sexes. The ATP-III, Ferranti, IDF, AHA and WHO criteria agree in less than 2% when identifying MS in the same group of adolescents.

## INTRODUCTION

As of 2020, the global prevalence of metabolic syndrome (MetS) in adolescents was 4.8%, representing 21.3 to 63.0 million; prevalence was even higher (7.0%) in low-income countries ^1^. The weighted prevalence was estimated from studies that used different diagnostic criteria; this lack of consensus is due to several reasons. First, some criteria extrapolate adult cut-off points. In addition, waist circumference is used to determine central obesity, but it varies according to the percentiles calculated with data from specific populations. Each component of MetS is dichotomous in measurement, which does not reflect the continuous quantitative nature of the original measures. Most criteria assume that their components contribute equally, that is, they define MetS by the number of components. Finally, adolescents of different races have different metabolic characteristics, which complicates having a single definition difficult ^2^.

In Peru, the frequency of MetS in adolescents in high Andean areas varies between 3.2% ^3^ and 4.6% ^4^, both figures were estimated using the Third Adult Treatment Panel (ATP-III) criteria modified by Cook. Another Peruvian study on children and adolescents with obesity attended in a specialized center in Lima found a prevalence of 22.3% ^5^. This study applied the International Diabetes Federation (IDF) criteria. ATP-III and IDF use blood pressure, some indicator of obesity, HDL cholesterol, triglycerides and serum glucose as components, which are the most frequently used components in cross-sectional studies to measure MetS in the population living at high altitudes ^6^.

The IDF, ATP-III and Ferranti criteria have been found to estimate different magnitudes of MetS in children and adolescents ^7^. These differences may be due to the particularities of the individuals in the sample of each study, such as ethnicity, eating habits and physical activity, socioeconomic status, age, among other variables; however, the fundamental element is the diagnostic criterion applied ^8^. Criteria with lax cut-off points generate higher prevalence of the MetS components; the definition of the presence of a component based on specific percentiles for age, sex and race, constructed from data from the reference population, also cause higher prevalence ^9^.

The concordance of different criteria for MetS in adolescents has been evaluated in several countries ^(^^10^^-^^15^; however, these studies were conducted at altitudes of less than 2000 meters above sea level (masl). The frequency of MetS has been reported to be lower at high altitudes ^16^^,^^17^, and the presence of its components may vary; for example, reports show a higher frequency of hypertriglyceridemia in Ecuador ^18^, as well as a lower frequency of hypercholesterolemia and hyperglycemia ^16^. A study on Tibetans reported higher blood pressure levels ^19^ and frequency of hypertension at higher altitudes ^17^. The lower frequency of MetS at high altitude could be explained by the inverse correlation between body mass index (BMI) and altitude above sea level, reflected in a lower prevalence of obesity and overweight in these populations ^20^; and by the lower frequency of dyslipidemia at high altitudes ^16^^,^^17^.

Therefore, there is no information available on the concordance of the different criteria for MetS in adolescents living in high Andean areas. In Peru, a study on adults over 40 years of age living at 3635masl found that the IDF and ATP-III criteria had a concordance index of 0.775 ^21^. Because of this knowledge gap and the difficulties in measuring MetS in high Andean areas, this study aimed to determine the concordance between five diagnostic criteria for MetS among adolescents living in a high Andean area of Peru.

**KEY MESSAGES**

Motivation for the study. There are several criteria for metabolic syndrome in adolescents, each reporting different prevalence rates and not necessarily coinciding with each other.

Main findings. We studied school children from the city of Cajamarca at 2750 meters above sea level. The five criteria for metabolic syndrome coincided in six of the 397 (1.5%) adolescents. The criteria generated prevalence rates ranging from 3.0% to 17.1%. The criteria with near perfect concordance were those from the American Heart Association criteria and those modified by Cook.

Implications. The diagnosis of metabolic syndrome in adolescents is complex, even more so among those residing at high altitudes.

## MATERIALS AND METHODS

### Design and scope of the study

We conducted a cross-sectional observational study with secondary data from pre-experimental research ^4^. The primary study was conducted in the city of Cajamarca, located at 2750 masl in northwestern Peru. This study evaluated the changes in the proportion of MetS components before and after an educational intervention based on healthy lifestyles. The primary study was conducted in two public educational institutions. In 2019, the “Juan XXIII” school, which is exclusive for women, had 1666 students in secondary education, while the “San Ramón” school, exclusive for men, had 1597 students. Both institutions are located in an urban area, and their students belong to families in the urban-marginal area of the city and nearby rural communities.

The sample size of the original study was calculated to contrast a hypothesis of comparison of proportions in paired samples; this calculation was made independently for each school. In each school, the sections (classrooms) were considered as the primary sampling unit, and we applied stratified random sampling to select them. The planned sample size for each school was 196 participants. Data collection was conducted between May and June 2019.

### Population and sample

The eligibility criteria for the original study were: to be a high school student in one of the two public schools in the district of Cajamarca, and to have provided informed assent (from the child) as well as informed consent from the mother, father or guardian on behalf of the child. Pregnant women and students with physical limitations that made anthropometry impossible were excluded from the study ^4^. In this study, a total of 388 students completed the baseline and final measurements.

We applied the following criteria during our analysis: having participated in the baseline measurement of the original study, having provided informed assent and parental informed consent for future use of the information, and being younger than 18 years of age. A total of 397 adolescents met these inclusion criteria. This number is higher than in the original study, as there were no exclusions for not completing the intervention or the post-intervention measurement.

### Metabolic syndrome criteria

Five criteria for MetS were used. The first is the Cook modified ATP-III criteria, which was adapted for adolescents aged 12 to 19 years from the criteria used in adults. Cook’s study was conducted with data from adolescents in the United States of America (USA) who participated in the Third National Health and Nutrition Examination Survey (1988 to 1994) (NHANES III). This sample included Mexican Americans ^22^. The second criterion was that of Ferranti *et al*. ^23^, which was constructed with data from NHANES III and, unlike the ATP-III criteria adapted by Cook, used lower cut-off points for waist circumference, triglycerides, and HDL cholesterol.

The third was the IDF criterion ^24^, developed by consensus of experts in MetS and pediatrics. This criterion establishes cut-off points for three groups (6 to <10, 10 to <16, and ≥16 years), in which abdominal obesity is a mandatory component, a characteristic that differentiates it from the other four criteria. The fourth was the criterion from the American Heart Association (AHA), which is based on the AHA criteria for adults, but uses pediatric population reference values for blood pressure, waist circumference, and HDL cholesterol ^25^, that is, three of the five components use percentiles. The fifth was the World Health Organization (WHO) criterion modified for children and adolescents. This criterion uses reference values for BMI instead of waist circumference and defines hypertension at values above the 95th percentile for age and sex. The cut-off points for each component of the five criteria are shown in Table 1.

**Table 1:** Criteria and cutoff points for each component of metabolic syndrome in adolescents.

Component			Metabolic syndrome criteria		
	ATP III (modified by Cook)	De Ferranti	IDF	AHA	WHO
Definition of Metabolic Syndrome	3 or more risk factors	3 or more risk factors	Central obesity plus 2 risk factors	3 or more risk factors	3 or more risk factors
Body mass index	Does not apply	Does not apply	Does not apply	Does not apply	> P95 ^a^ (age/sex)
Waist circumference (cm) ^a^	≥ P90 (age/sex)	> P75 (age/sex)	<16 years: ≥ P90 (age/sex) ≥ 16 years (men): ≥ 94 ≥ 16 years (women): ≥ 80	≥ P90 (age/sex)	Does not apply
Fasting glucose (mg/dL)	≥ 110	≥ 110	≥ 100	≥ 100	≥ 110
Triglycerides (mg/dL)	≥ 110	≥ 100	≥ 150	≥ 110	> 136
HDL Colesterol (mg/dL) ^b^	≤ 40	< 50 < 45 men from 15 to 19 years	< 16 years: < 40 ≥ 16 years (men): < 40 ≥ 16 years (women): < 50	≤ P10 for race and sex	< 35
Systolic blood pressure (mmHg) ^c^	≥ P90 (age/sex)	≥ P90 (age/sex)	≥ 130	≥ P90 (age/sex)	≥ P95 (age/sex)
Diastolic blood pressure3 (mmHg) ^c^	≥ P90 (age/sex)	≥ P90 (age/sex)	≥ 85	≥ P90 (age/sex)	Does not apply

ATP-III: Third Adult Treatment Panel, IDF: International Diabetes Federation, AHA: American Heart Association, WHO: World Health Organization, P95: 95th percentile, P90: 90th percentile, P80: 80th percentile, P75: 75th percentile, P10: 10th percentile.

a Percentiles for waist circumference and body mass index were obtained from the Technical Guide for the anthropometric nutritional assessment of adolescents. Peruvian Ministry of Health, 2015.

b The percentile for HDL cholesterol values were obtained from Cook *et al*. Growth Curves for Cardio-Metabolic Risk Factors in Children and Adolescents. J Pediatr. 2009;155(3):S6.e15-S6.e26. doi: 10.1016/j.jpeds.2009.04.051.

c The percentiles for systolic and diastolic blood pressure were obtained from the calculator: https://www.msdmanuals.com/es-pe/professional/pages-with-widgets/calculadoras-cl%c3%adnicas?mode=list.

### Measurement of variables

Abdominal perimeter, height and weight were measured by a nurse trained and certified as an anthropometrist in the Peruvian technical standard ^26^. Blood pressure was measured by six nurses, who took the measurements of both pressures (systolic and diastolic) on three occasions between 8 and 9 am, the time interval between each measurement was three minutes. For the analysis, we considered the average of the three measurements. Riester exacta® aneroid sphygmomanometers with Velcro cuffs for small adults and Riester Duplex® stethoscopes were used. During the measurement, the student was seated with the arm flexed in a suitable position (resting on the table) and with the inflatable cuff covering two thirds of the length and circumference of the arm.

Venipuncture was performed, with the student fasting, by a medical technologist in order to obtain blood samples. The amount of whole blood collected was 5 mL in a polyethylene terephthalate tube with coagulant activator and gel. The samples were transferred to a private laboratory within one hour after sampling; they were then centrifuged and separated into aliquots to be processed on the same day of blood collection.

The laboratory tests included measuring glucose levels, total cholesterol and triglycerides in serum (mg/dL). These procedures were performed with the enzymatic method and spectrophotometer readings. HDL cholesterol was measured by the colorimetric method without precipitation. The reagents used for all the tests were of the Wiener brand, and the automatic biochemical analyzer used was the Wiener model CB 400i.

### Statistical analysis

The Kolmogorov-Smirnov test was used to evaluate whether age, systolic blood pressure, diastolic blood pressure, height, weight, waist circumference, HDL cholesterol, BMI, triglycerides, and fasting glucose were normally distributed. The mean and standard deviation or median with interquartile range (IQR) were used according to the type of distribution of the quantitative variable. The difference in means and medians between women and men was evaluated using Student’s t-test and Mann-Whitney U test, respectively. The components of the MetS for each diagnostic criterion are presented as frequencies and proportions expressed as percentages, according to sex and the whole sample. In addition, the proportion of MetS and their 95% confidence intervals (95% CI) were estimated.

Cohen’s Kappa coefficient was estimated in order to evaluate the concordance between the five criteria. The following levels were considered ^27^: slight (0.01 to 0.20), acceptable (0.21 to 0.40), moderate (0.41 to 0.60), substantial (0.61 to 0.80) and almost perfect (0.81 to 1.00). The percentage of total agreement between the criteria was calculated as the sum of concordance in positive and negative results among the total sample. The concordance evaluation was performed for the whole sample and according to sex. The statistical analysis was performed in Jamovi 2.2.5, and a value of p<0.05 was considered to be statistically significant.

### Ethical aspects

The original study was approved by the Research Ethics Committee of the Universidad Nacional de Cajamarca (code 009-2019). In this research, authorization for the future use of the data was requested by means of the informed consent. The database we used had been anonymized since the date of completion of the formal statistical analysis of the original study (August 2021).

## RESULTS

### Sample characteristics

We analyzed data from 397 adolescents, of whom 196 were male (49.4%). The median age was 14 years, half were 13 to 16 years old. The median systolic blood pressure, diastolic blood pressure, height and weight were higher in males than in females. On the other hand, median HDL cholesterol, BMI and triglycerides were higher in females than in males (Table 2).

**Table 2:** Characteristics of adolescents enrolled in two educational institutions in a high Andean area of Peru.

Variable	Total (N = 397)		Women ^b^ (n = 201)		Men ^b^ (n = 196)		p-value ^a^
	Median	IQR	Median	IQR	Median	IQR	
Age (years)	14	13 - 16	14	13 - 16	14	13 - 16	0,032
Mean systolic pressure (mmHg)	100.0	90.0 - 103.3	93.3	90.0 - 102.0	100	93.3 - 107.0	<0.01
Mean diastolic pressure (mmHg)	63.3	60.0 - 70.0	60.0	60.0 - 70.0	66.7	60.0 - 70.0	<0.01
Height (m)	1.52	1.47 - 1.58	1.49	1.46 - 1.54	1.57	1.50 - 1.62	<0.01
Weight (kg)	50.8	44.4 - 56.8	49.5	44.0 - 55.1	53.0	46.2 - 58.9	<0.01
Waist circumference (cm)	72.0	68.0 - 78.0	71.0	67.0 - 77.0	73.0	68.0 - 78.3	0.110
HDL (mg/dL)	38.0	33.0 - 43.0	38.0	34.0 - 44.0	36.0	30.0 - 42.0	<0.01
BMI (kg/m^2^)	21.5	19.7 - 23.8	21.9	20.1 - 24.0	21.2	19.2 - 23.5	0.028
Triglycerides (mg/dL)	98.0	76.0 - 134.0	103.0	79.0 - 142.0	94.5	72.8 - 126.0	0.035
Glucose (mg/dL)	80.0	74.0 - 86.0	80.0	73.0 - 85.0	81.0	74.0 - 86.0	0.506

a p-value corresponding to the Mann Whitney U test.

b Women were enrolled in the “Juan XXIII” school, men were enrolled in the “San Ramón” school.

BMI: body mass index; HDL cholesterol: high-density lipoprotein cholesterol; IQR: interquartile range; HDL cholesterol: high-density lipoprotein cholesterol.

### Components of the metabolic syndrome

Regarding the central obesity component, the Ferranti criteria identified the highest proportion of adolescents (n = 80; 20.2%). The IDF and AHA criteria identified one case with glucose above the cut-off point. Low HDL cholesterol was the most frequent MetS component, ranging from 34.8% with the WHO criteria to 90.4% with the Ferranti criteria. The latter criterion also identified the highest proportion of hypertriglyceridemia (48.9%). The ATP-III, Ferranti and AHA criteria each identified 25 hypertensive adolescents (Table 3).

**Table 3:** Frequency of metabolic syndrome components according to five diagnostic criteria.

Criterion ^a^	Sex ^b^		Total
	Men (n = 196)	Women (n = 201)	
	n (%)	n (%)	n (%)
ATP - III			
Metabolic syndrome	11 (5.6)	5 (2.5)	16 (4.0)
Central obesity	5 (2.6)	7 (3.5)	12 (3.0)
Hyperglycemia	0 (0)	0	0
Hypertriglyceridemia	68 (34.7)	89 (44.3)	157 (39.5)
Low HDL	135 (68.9)	123 (61.2)	258 (65.0)
Arterial hypertension	19 (9.7)	6 (3.0)	25 (6.3)
De Ferranti			
Metabolic syndrome	39 (19.9)	29 (14.4)	68 (17.1)
Central obesity	43 (21.9)	37 (18.4)	80 (20.2)
Hyperglycemia	0	0	0
Hypertriglyceridemia	86 (43.9)	108 (53.7)	194 (48.9)
Low HDL	181 (92.3)	178 (88.6)	359 (90.4)
Arterial hypertension	19 (9.7)	6 (3.0)	25 (6.3)
IDF			
Metabolic syndrome	5 (2.6)	7 (3.5)	12 (3.0)
Central obesity	8 (4.1)	20 (10.0)	28 (7.1)
Hyperglycemia	0	1 (0.5)	1 (0.3)
Hypertriglyceridemia	31 (15.8)	41 (20.4)	72 (18.1)
Low HDL	125 (63.8)	127 (63.2)	252 (63.5)
Arterial hypertension	0	0	0
AHA			
Metabolic syndrome	9 (4.6)	6 (3.0)	15 (3.8)
Central obesity	5 (2.6)	7 (3.5)	12 (3.0)
Hyperglycemia	0	1 (0.5)	1 (0.3)
Hypertriglyceridemia	68 (34.70)	89 (44.3)	157 (39.5)
Low HDL	89 (45.4)	88 (43.8)	177 (44.6)
Arterial hypertension	19 (9.7)	6 (3.0)	25 (6.3)
OMS			
Metabolic syndrome	11 (5.6)	3 (1.5)	14 (3.5)
Hyperglycemia	0	0	0
Hypertriglyceridemia	42 (21.4)	55 (27.4)	97 (24.4)
Low HDL	85 (43.4)	53 (26.4)	138 (34.8)
Arterial hypertension	0	0	0
BMI > P95	20 (10.2)	10 (5.0)	30 (7.6)

HDL: high-density lipoprotein cholesterol; HT: hypertension; BMI: body mass index; P95: 95th percentile; ATP-III: Third Adult Treatment Panel; IDF: International Diabetes Federation; AHA: American Heart Association; WHO: World Health Organization.

a The cut-off points for each component of metabolic syndrome in adolescents have been defined in Table 1.

b Women were enrolled in the “Juan XXIII” school and men were enrolled in the “San Ramón” school.

### Frequency of metabolic syndrome

The Ferranti criterion classified 17.1% of adolescents as MetS (95% CI: 13.4 to 20.8), the ATP-III criterion identified 4.0% (95% CI: 2.1 to 6.0), the AHA criterion 3.8% (95% CI: 1.9 to 5.7), the WHO criterion 3.5% (95% CI: 1.7 to 5.3), and the IDF criterion identified the fewest cases (3.0%; 95% CI: 1.3 to 4.7). The Ferranti, ATP-III, AHA and WHO criteria identified a higher proportion of cases of MetS among men (Table 3).

### Concordance of criteria

The highest percentage of total concordance was found between the AHA and ATP-III criteria (99.2%). The IDF criteria had percentages of concordance of 97.0% and 97.7% with the AHA and WHO, respectively. The proportion of concordance between the criteria differed between men and women (Table 4).

**Table 4:** Number of concordant cases and percentage of concordance between five diagnostic criteria for metabolic syndrome.

Group	ATP III				De Ferranti				IDF				AHA			
	+	-	Total	%	+	-	Total	%	+	-	Total	%	+	-	Total	%
Total																
De Ferranti	17	329	346	87.2												
IDF	7	375	382	96.2	11	328	339	85.4								
AHA	15	379	394	99.2	16	329	345	86.9	8	377	385	97.0				
OMS	8	375	383	96.5	13	329	342	86.1	8	380	388	97.7	8	376	384	96.7
Men																
De Ferranti	11	157	168	85.7												
IDF	4	184	188	95.9	5	157	162	82.7								
AHA	9	185	194	99.0	9	157	166	84.7	4	186	190	96.9				
OMS	6	181	187	95.4	10	157	167	85.2	5	186	191	97.4	6	183	189	96.4
Women																
De Ferranti	6	172	178	88.6												
IDF	3	191	194	96.5	6	171	177	88.1								
AHA	6	194	200	99.5	7	172	179	89.1	4	191	195	97.0				
OMS	2	194	196	97.5	3	172	175	87.1	3	194	197	98.0	2	193	195	97.0

+: concordance in positive cases, -: concordance in negative cases, %: percentage of total concordance.

ATP-III: Third Adult Treatment Panel, IDF: International Diabetes Federation, AHA: American Heart Association, WHO: World Health Organization.

**Figure 1:**
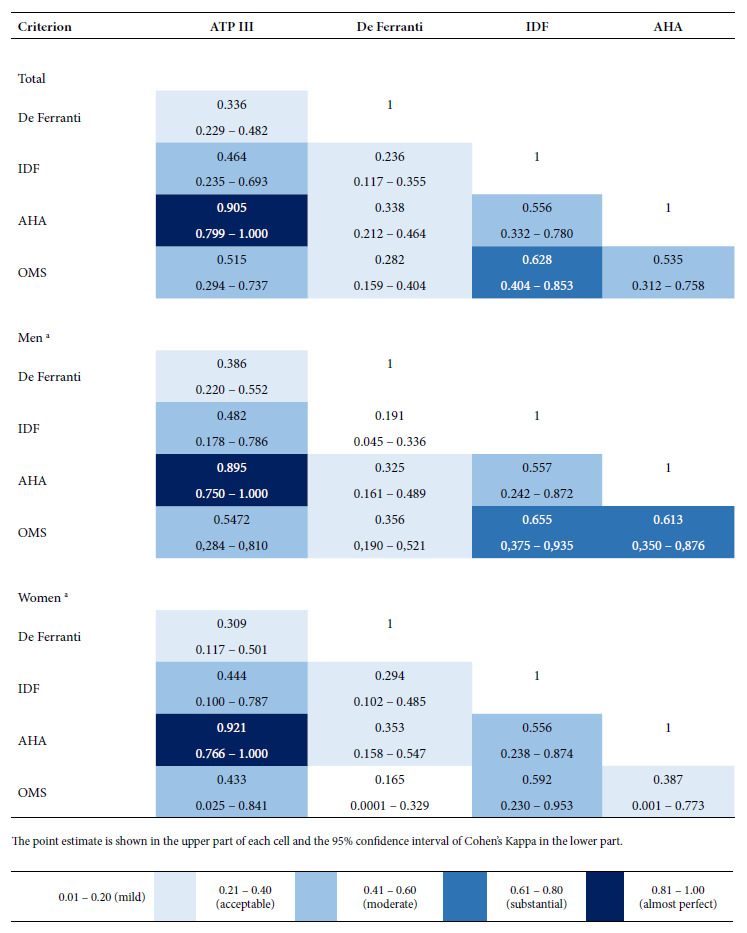
Estimation of the concordance between five diagnostic criteria of metabolic syndrome among schoolchildren from two public educational institutions in a high Andean district (Cajamarca, Peru).

An “almost perfect” concordance was found between the AHA and ATP-III criteria (k = 0.905); this level of agreement was observed in men (k = 0.895) and in women (k = 0.921). “Substantial” concordance was found between the WHO and IDF criteria (k = 0.628); however, this level of concordance was not observed among women (k = 0.592). A “moderate” level of concordance was found between the IDF criteria and the ATP-III and AHA criteria, as well as between the WHO criteria and the ATP-III and AHA criteria. The Ferranti criterion had an “acceptable” level of concordance with the other criteria (Figure 1).

The five criteria coincided in classifying 6 adolescents (1.5%) as MetS, four criteria coincided in 3 (0.8%), three criteria in 9 (2.3%) and two criteria in 6 (1.5%). Finally, 45 (11.3%) adolescents were identified as MetS by one of the criteria used.

## DISCUSSION

The AHA and ATP-III criteria had “almost perfect” concordance for identifying MetS among adolescents residing in a high Andean area of Peru; on the other hand, the Ferranti criteria had the lowest levels of agreement with respect to the IDF, AHA, WHO and ATP-III criteria. The estimated concordance coefficient between the AHA and ATP-III criteria was higher than what was found in a study of 1004 European adolescents aged 12.5 to 17 years. In that study, the AHA and ATP-III criteria reached a kappa between 0.73 and 0.80; however, as in our study, this coefficient was the highest among those generated by the comparison of five different criteria (ATP-III, AHA, WHO, IDF and those of Jolliffe & Janssen) ^14^.

The level of concordance between AHA and ATP-III could be explained by the fact that both criteria only differ in the definition of one component. The ATP-III criterion modified by Cook considers low HDL cholesterol when it is lower than 40 mg/dL, whereas the AHA uses values equal to or below the 10th percentile for race and sex. Due to the absence of reference values for Peruvian adolescents or residents of high Andean areas, we used the percentile values defined by a national health and nutrition survey of the United States of America ^28^, which Cook used to define the value of 40 mg/dL for HDL cholesterol.

“Substantial” concordance was found between the WHO and IDF criteria; only one study has reported concordance between these criteria. In European adolescents, a kappa coefficient between 0.29 and 0.38 was found ^14^, which, according to the cutoffs applied in our study, corresponds to an “acceptable” agreement. The WHO criterion is different from the others: i) it uses BMI instead of waist circumference; ii) it defines hypertension at values equal to or higher than the 95th percentile of blood pressure; and iii) it uses the most restrictive cutoff to define low HDL cholesterol (< 35 mg/dL). Despite these differences, we found a higher concordance with the IDF criteria than previously reported. One possible explanation is that both criteria did not identify cases of arterial hypertension, another explanation may be the fact that both use the highest and closest cutoffs for defining hypertriglyceridemia.

Concordance between the ATP-III and IDF criteria has been the most studied among adolescents from different countries, with very different levels of concordance. In Latin America, the total concordance between these criteria was 97.5%, and the kappa coefficient was 0.95 among 241 adolescents aged 10 to 14 years in the state of Paraná in Brazil ^12^. Another study conducted on 1200 adolescents aged 12 to 17 years in the state of Santa Cruz do Sul in Brazil estimated a kappa of 0.53 ^15^. A kappa of 0.39 was estimated in 851 adolescents aged 10 to 18 years in the city of Medellín in Colombia ^10^; while a kappa of 0.28 was estimated in 916 children and adolescents aged 9 to 18 years in the city of Mérida in Venezuela, ^29^. Among the studies in Europe, one conducted on 379 Spanish adolescents aged 12 to 16.9 years found a kappa of 0.82 ^30^. Another study conducted on adolescents aged 10 to 16 years in Italy reported a kappa of 0.71 ^31^, while a kappa of 0.47 was estimated in 1004 adolescents living in 10 European cities ^14^.

The different concordance coefficients between the ATP-III and IDF criteria can be explained by the fact that although they use the same components, the IDF criterion requires the presence of central obesity, a component that, in turn, is determined differently from ATP-III. The IDF criterion applies a cut-off determined by the 90th percentile for those under 16 years of age, while using 94 and 80 cm as cut-off points for males and females 16 years and older, respectively. Other differences are the cut-off points for defining hyperglycemia and hypertriglyceridemia, the cut-off for glycemia in IDF is lower compared to ATP-III; while the cut-off for triglyceride concentration is higher in ATP-III. Finally, IDF uses fixed cutoff points (≥130/85 mmHg) to define arterial hypertension, whereas ATP-III uses percentiles. Based on these differences, the ATP-III criterion classifies a higher proportion of adolescents as MetS when compared to IDF; this has been reported in Brazil ^12^, Spain ^30^, Colombia ^10^, several European countries ^14^, and by our study.

The Ferranti criterion identified MetS in a higher proportion of adolescents (17.1%), on average, it identified three to four times more cases than the other criteria. This finding has been reported by other studies, for example, in Brazil, the Ferranti criterion identified 17.4% of adolescents as having MetS, higher than that identified by the ATP-III criteria modified by Cook (3.3%) and IDF (1.7%) ^12^. In Colombia, Ferranti’s criteria found 11.4% of MetS, also higher than the ATP-III criteria modified by Cook (3.8%) and IDF (0.9%) ^10^. This finding may be due to the less restrictive cut-off points of the Ferranti criteria for defining central obesity, hypertriglyceridemia and low cholesterol levels.

We found that low HDL cholesterol was the component most frequently identified by all the criteria. The Ferranti criterion defined this component in 90.4% of adolescents, followed by the ATP-III criteria modified by Cook (65%) and the IDF (63.5%). The second most frequent component was hypertriglyceridemia; likewise, the Ferranti criteria identified 48.9% of adolescents with this condition, followed by the ATP-III and AHA criteria. This pattern is similar to that found in adolescents in Colombia ^10^, but differs from that found in other adolescent populations. For example, the most frequent component in Spanish adolescents was abdominal obesity ^13^^,^^30^, as in Mexican-American adolescents in the USA ^32^; while in Brazil, the most frequent component was arterial hypertension ^12^^,^^15^.

Several studies show a particular lipid profile in adolescents and adults in the Andes, characterized by high serum triglyceride levels, low HDL cholesterol levels and hypercholesterolemia ^3^^,^^33^, such profile has also been found in Tibetan adults (3660 masl) ^34^, while the prevalence of hypertriglyceridemia or low HDL cholesterol was 9.1% in adults residing above 2000 masl in China ^17^. It is reported that dyslipidemias may be a frequent problem in adolescents. The high frequency of dyslipidemias found in adolescents residing at almost 3000 masl could be explained by genetic factors ^35^, and the influence of metabolic and hormonal adaptations to altitude; however, this hypothesis requires specific research.

Only the IDF and AHA criteria identified one case of elevated glycemia, both criteria have a cut-off point of 100 mg/dL. The low frequency of this component has been previously reported, with a cutoff of 110 mg/dL in adolescents in the city of Cajamarca ^3^, as well as in other studies on the concordance of MetS criteria in Colombia ^10^, Brazil ^11^^,^^15^ and Spain ^13^. This finding is consistent with the adaptations of the cellular response to chronic hypoxia at high altitudes ^17^^,^^36^, and with the low frequency of abdominal obesity and the consequent low risk of insulin resistance.

What we described in this concordance analysis shows how complex the diagnosis of MetS in adolescents is. These results have implications for future research in adolescents living at high altitude, as they show how crucial the selection of the diagnostic criterion is and how this decision can affect the frequency estimates of MetS. In general, Ferranti’s criterion classifies a higher proportion of adolescents as MetS; this finding does not imply better diagnostic performance; in fact, it is the criterion with the lowest concordance with the others. Secondarily, our analysis identified the high frequency of hypertriglyceridemia and low HDL cholesterol as a potential public health problem. Therefore, the diagnosis of MetS in adolescents requires criteria that considers the continuous nature of its components and the construction of scores that define cardiometabolic risk based on population-based and representative data at different altitudinal levels. Adequately defining the presence of each component will make it possible to improve the identification of MetS and to establish intervention and prevention strategies in adolescents with or without MetS.

The study has some limitations. Percentile values for systolic and diastolic blood pressure and HDL cholesterol were not available, so we used data available from NHANES III conducted in the United States. Since this was a secondary source study, no *a priori* calculation of the sample size was made in order to estimate concordance coefficients. The WHO criterion assesses glycemic homeostasis by means of insulin levels, glucose intolerance and fasting glucose; only the latter was used in this analysis. Finally, several criteria use arbitrary cut-off points to define the presence of a component, especially for hyperglycemia and hypertriglyceridemia; such cut-offs may vary and require reevaluation when the lifestyles and risk factors of populations change, as well as laboratory procedures. Besides, our results should be interpreted with caution. We studied a single district located in the Peruvian Andes; therefore, in view of the diverse altitudinal levels, lifestyles, and ethnicities, our results are not representative for adolescents living in the Peruvian Andes. Our results do not evaluate or conclude which criterion has the best diagnostic performance for MetS; future studies should be conducted to evaluate criterion validity compared to a reference diagnosis.The complexity of this lies in the fact that to date there is no consensus on the diagnosis of MetS in adolescents.

In conclusion, the five criteria agree by less than 2% when identifying the same group as having MetS, which produces different prevalence estimates in adolescent schoolchildren living in a high Andean area in the city of Cajamarca, Peru. The AHA and ATP-III criteria modified by Cook had “almost perfect” concordance; this level of concordance was found for both sexes. The Ferranti criterion identified MetS in 17.1% of adolescents; this criterion identified three to four times more MetS cases than the others.
