# Supplementary material for: Concordance between five criteria of metabolic syndrome in teenagers from a Peruvian high andes region
Source: Rev Peru Med Exp Salud Publica. 2023 Jun 30;40(2):150–60. doi: 10.17843/rpmesp.2023.402.12546 (PMC10953662; doi:10.17843/rpmesp.2023.402.12546)
Supplement: Translation

# Translation: Concordancia entre cinco criterios de síndrome metabólico en adolescentes de una zona altoandina del Perú

**Authors:** Franco Romaní-Romaní, Luis Fernando Pachacama Ramirez, Juan Diego Pichihua Grandez, Diego Maximiliano Guevara Rodríguez, Viviana Cornejo Luyo, Christian Eduardo Sheen Vargas, Juana Aurelia Ninatanta-Ortiz, Martha Vicenta Abanto Villar, Katia Maribel Pérez Cieza, Rosa Ricardina Chávez Farro, Segunda Aydeé García Flores

**Affiliations:** 1 Facultad de Medicina Humana, Universidad de Piura, Lima, Perú.; 2 Escuela Académico Profesional de Enfermería, Facultad Ciencias de la Salud, Universidad Nacional de Cajamarca, Cajamarca, Perú.

**Keywords:** Síndrome Metabólico, Adolescente, Diagnóstico, Perú

## Abstract

**Objetivo.:**

Determinar la concordancia entre cinco criterios diagnósticos de síndrome metabólico (SM) entre adolescentes residentes de una zona altoandina del Perú.

**Materiales y métodos.:**

Se realizó un estudio transversal con datos secundarios de un estudio de intervención en dos escuelas públicas en el año 2019. Participaron 397 adolescentes que residen en la ciudad de Cajamarca ubicada en la región andina del Perú. Se aplicaron cinco criterios diagnósticos: *Third Adult Treatment Panel* (ATP-III) modificados por Cook, de la *Federación Internacional de Diabetes* (IDF), de la *American Heart Association* (AHA), de Ferranti, y de la Organización Mundial de Salud (OMS). Se estimaron las prevalencias puntuales y de intervalo con los cinco criterios. Se estimó el coeficiente de concordancia de Kappa con su respectivo intervalo de confianza al 95% (IC 95%).

**Resultados.:**

El criterio de Ferranti identificó un 17,1% (IC 95%: 13,4 a 20,8) de adolescentes con SM, seguido del criterio de la ATP-III con 4,3% (IC 95%: 2,3 a 6,3); los demás criterios identificaron una menor frecuencia. La mejor concordancia fue encontrada entre los criterios de la AHA y la ATP-III (k = 0,905), los criterios de la OMS e IDF tuvieron un coeficiente de 0,628. Los cinco criterios coincidieron en clasificar como SM a seis adolescentes (1,5%).

**Conclusiones.:**

Los criterios de la AHA y la ATP-III modificados por Cook tuvieron una concordancia casi perfecta, que se mantuvo en ambos sexos. Los criterios ATP-III, de Ferranti, IDF, AHA y OMS concuerdan en menos del 2% en identificar SM en el mismo grupo de adolescentes.

## INTRODUCCIÓN

Al 2020, la prevalencia global de síndrome metabólico (SM) en adolescentes fue 4,8%, lo cual representó de 21,3 a 63,0 millones de afectados; entre países de bajos ingresos, la prevalencia fue aún mayor (7,0%) ^1^. Estas prevalencias ponderadas fueron estimadas de estudios individuales que usaron diferentes criterios diagnósticos, esta falta de consenso se da por varias razones. Primero, algunos criterios extrapolan puntos de corte de adultos; segundo, la circunferencia de cintura, usada para determinar la obesidad central, varía en función de percentiles calculados con datos de poblaciones específicas; tercero, cada componente del SM es de medición dicotómica, lo cual no refleja la naturaleza cuantitativa continua de las medidas originales; cuarto, la mayoría de criterios asumen que sus componentes contribuyen igualmente, es decir, definen SM con el número de componentes presentes; y quinto, los adolescentes de diferentes razas presentan características metabólicas diferentes que dificultan una definición única ^2^.

En Perú, la frecuencia de SM en adolescentes de zonas altoandinas varía entre 3,2% ^3^ y 4,6% ^4^, ambas prevalencias fueron estimadas con los criterios del *Third Adult Treatment Panel* (ATP-III) modificados por Cook. Otro estudio peruano, en niños y adolescentes con obesidad atendidos en un centro especializado de Lima encontró una prevalencia de 22,3% ^5^. Este estudio aplicó los criterios de la *International Diabetes Federation* (IDF). ATP-III e IDF usan como componentes a la presión arterial, algún indicador de obesidad, colesterol HDL, triglicéridos y glucosa sérica; los cuales son los componentes más frecuentemente usados por estudios transversales para medir SM en población residente en elevadas altitudes ^6^.

En niños y adolescentes se ha encontrado que los criterios de la IDF, de la ATP-III y de Ferranti estiman diferentes magnitudes de SM ^7^. Estas diferencias pueden deberse a las particularidades de los individuos que conforman la muestra de cada estudio, como la etnicidad, hábitos de alimentación y actividad física, estado socioeconómico, edad, entre otras variables; sin embargo, el elemento fundamental es el criterio diagnóstico aplicado ^8^. Los criterios con puntos de corte laxos generan mayores prevalencias de los componentes del SM; la definición de presencia de un componente en base a percentiles específicos para edad, sexo y raza, construidos de datos provenientes de la población de referencia, también generan mayores prevalencias ^9^.

En diversos países, se ha evaluado la concordancia de diferentes criterios de SM en adolescentes ^10^^-^^15^, sin embargo, dichos estudios fueron realizados a menos de 2000 metros sobre el nivel del mar (msnm). Se reporta que, la frecuencia del SM es menor en elevadas altitudes ^16^^,^^17^, además, la presencia de sus componentes puede variar; por ejemplo, en Ecuador, se ha observado una mayor frecuencia de hipertrigliceridemia ^18^ y menor frecuencia de hipercolesterolemia e hiperglicemia ^16^. En tibetanos, se ha reportado mayores niveles de presión arterial ^19^ y frecuencia de hipertensión a mayor altitud ^17^. La menor frecuencia del SM en altura se explicaría por la correlación inversa entre el índice de masa corporal (IMC) y la altitud sobre el nivel del mar, reflejada en una menor prevalencia de obesidad y sobrepeso en estas poblaciones ^20^; y por la menor frecuencia de dislipidemias a elevadas alturas ^16^^,^^17^.

En el contexto descrito, no se dispone de información sobre la concordancia de los diferentes criterios de SM en adolescentes que residen en zonas altoandinas. En Perú, hay un antecedente entre adultos mayores de 40 años que viven a 3635 m s. n. m. y que encontró que los criterios de la IDF y de la ATP-III tuvieron un índice de concordancia de 0,775 ^21^. Debido a esta brecha en el conocimiento y las dificultades en la medición del SM en zonas altoandinas, este estudio tuvo como objetivo determinar la concordancia entre cinco criterios diagnósticos de SM entre adolescentes residentes de una zona altoandina del Perú.

**MENSAJES CLAVE**

Motivación para realizar el estudio. Existen diversos criterios para el síndrome metabólico en adolescentes, cada uno genera diferentes prevalencias y no necesariamente coinciden entre ellos.

Principales hallazgos. Estudiamos escolares de la ciudad de Cajamarca a 2750 metros sobre el nivel del mar, los cinco criterios de síndrome metabólico coincidieron en seis de los 397 (1,5%) adolescentes. Los criterios generaron prevalencias que varían de 3,0% a 17,1%, y aquellos con un acuerdo casi perfecto fueron los criterios de la Asociación Americana del Corazón y los modificados por Cook.

Implicancias. El diagnóstico del síndrome metabólico en adolescentes es complejo, más aún entre aquellos que residen a elevadas altitudes.

## MATERIALES Y MÉTODOS

### Diseño y ámbito de estudio

Se realizó un estudio observacional de corte transversal basado en datos secundarios de una investigación preexperimental ^4^. El estudio primario fue ejecutado en la ciudad de Cajamarca, ubicada a 2750 m s. n. m., al noroeste del Perú. Dicho estudio evaluó los cambios en la proporción de componentes del SM antes y después de una intervención educativa basada en estilos de vida saludable. El estudio primario fue ejecutado en dos instituciones educativas públicas. En el año 2019, el Colegio «Juan XXIII» exclusivo para mujeres tenía 1666 estudiantes de educación secundaria, mientras que el Colegio «San Ramón» para varones tenía 1597 alumnos. Ambas instituciones pertenecen a la zona urbana, y sus estudiantes provienen de familias de la zona urbano-marginal de la ciudad y de comunidades rurales cercanas.

El tamaño muestral del estudio original fue calculado para contrastar una hipótesis de comparación de proporciones en muestras pareadas, dicho cálculo se hizo de manera independiente para las escuelas intervenidas. En cada escuela, se consideró a las secciones (aulas) como unidad primaria de muestreo, para seleccionarlas se aplicó un muestreo aleatorio estratificado. El tamaño muestral planeado para cada escuela fue de 196 participantes. La recolección de datos fue realizada entre mayo y junio del 2019.

### Población y muestra

El estudio original tuvo como criterios de elegibilidad: ser estudiante de secundaria de alguno de los dos colegios públicos del distrito de Cajamarca, haber brindado el asentimiento informado por parte del menor y el consentimiento informado por parte de la madre, padre o apoderado. Las gestantes y estudiantes con limitación física que imposibilite la antropometría fueron excluidos del estudio ^4^. En dicho estudio, un total de 388 estudiantes completaron la medición basal y final.

Para el presente análisis, se aplicaron los siguientes criterios: haber participado en la medición basal del estudio original, haber brindado el asentimiento informado y que los padres hayan dado el consentimiento informado para el uso futuro de la información, y tener menos de 18 años. Un total de 397 adolescentes cumplieron estos criterios de inclusión. Este número es mayor al del estudio original, en vista que no hubo exclusiones por no completar la intervención o la medición posintervención.

### Criterios de síndrome metabólico

Se usaron cinco criterios para SM. El primero es el criterio de la ATP-III modificado por Cook, el cual fue adaptado para adolescentes de 12 a 19 años a partir de los criterios usados en adultos. El estudio de Cook fue realizado con datos de adolescentes de los Estados Unidos de Norteamérica (EE. UU.) que participaron en la Tercera Encuesta Nacional de Salud y Nutrición (1988 a 1994) (NHANES III, por sus siglas en inglés). Dicha muestra incluyó mexicoamericanos ^22^. El segundo criterio fue el de Ferranti *et al.*^23^, que fue construido con datos de la NHANES III y, a diferencia de los criterios ATP-III adaptados por Cook, se utilizan puntos de corte inferiores para la circunferencia de cintura, triglicéridos y colesterol HDL.

El tercero fue el criterio de la IDF ^24^, desarrollado por consenso de expertos en SM y pediatría. Este criterio establece puntos de corte para tres grupos (6 a <10, 10 a <16 y ≥16 años), en los cuales, la obesidad abdominal es un componente obligatorio, esta característica la diferencia de los otros cuatro criterios. El cuarto criterio fue de la Asociación Americana del Corazón (AHA, por sus siglas en inglés), que está basado en los criterios AHA para adultos, pero usa valores de referencia de población pediátrica para la presión arterial, circunferencia de cintura y colesterol HDL ^25^, es decir, tres de los cinco componentes usan percentiles. El quinto criterio de la Organización Mundial de la Salud (OMS) está modificado para niños y adolescentes. Este criterio utiliza valores de referencia para el IMC en lugar de la circunferencia de cintura y define la hipertensión arterial a los valores superiores al percentil 95 para edad y sexo. Los puntos de corte para cada componente de los cinco criterios se muestran en la Tabla 1.

**Tabla 1:** Criterios y puntos de corte para cada componente de síndrome metabólico en adolescentes.

Componente			Criterio de síndrome metabólico		
	ATP III (modificado por Cook)	De Ferranti	IDF	AHA	OMS
Definición de Síndrome Metabólico	3 o más factores de riesgo	3 o más factores de riesgo	Obesidad central más 2 factores de riesgo	3 o más factores de riesgo	3 o más factores de riesgo
Índice de masa corporal	No aplica	No aplica	No aplica	No aplica	> P95 ^a^ (edad/sexo)
Circunferencia de cintura (cm) ^a^	≥ P90 (edad/sexo)	> P75 (edad/sexo)	<16 años: ≥ P90 (edad/sexo) ≥ 16 años (varones): ≥ 94 ≥ 16 años (mujeres): ≥ 80	≥ P90 (edad/sexo)	No aplica
Glucosa en ayunas (mg/dL)	≥ 110	≥ 110	≥ 100	≥ 100	≥ 110
Triglicéridos (mg/dL)	≥ 110	≥ 100	≥ 150	≥ 110	> 136
Colesterol HDL (mg/dL) ^b^	≤ 40	< 50 < 45 varones de 15 a 19 años	< 16 años: < 40 ≥ 16 años (varón): < 40 ≥ 16 años (mujer): < 50	≤ P10 para raza y sexo	< 35
Presión arterial sistólica (mmHg) ^c^	≥ P90 (edad/sexo)	≥ P90 (edad/sexo)	≥ 130	≥ P90 (edad/sexo)	≥ P95 (edad/sexo)
Presión arterial diastólica (mmHg) ^c^	≥ P90 (edad/sexo)	≥ P90 (edad/sexo)	≥ 85	≥ P90 (edad/sexo)	No aplica

ATP-III: Third Adult Treatment Panel, IDF: International Diabetes Federation, AHA: American Heart Association, OMS: Organización Mundial de la Salud, P95: percentil 95, P90: percentil 90, P80: percentil 80, P75: percentil 75, P10: percentil 10.

a Los percentiles para la circunferencia de cintura e índice de masa corporal fueron obtenidos de la Guía Técnica para la valoración nutricional antropométrica de la persona adolescente. Ministerio de Salud del Perú, 2015.

b El percentil para los valores de colesterol HDL fueron obtenidos de Cook *et al*. Growth Curves for Cardio-Metabolic Risk Factors in Children and Adolescents. J Pediatr. 2009;155(3):S6.e15-S6.e26. doi: 10.1016/j.jpeds.2009.04.051.

c Los percentiles para la presión arterial sistólica y diastólica fueron obtenidos de la calculadora: https://www.msdmanuals.com/es-pe/professional/pages-with-widgets/calculadoras-cl%c3%adnicas?mode=list

### Medición de las variables

El perímetro abdominal, la talla y el peso fueron medidos por una enfermera entrenada y con certificación como antropometrista en la norma técnica peruana ^26^. La presión arterial fue medida por seis enfermeras, quienes realizaron las mediciones de ambas presiones (sistólica y diastólica) en tres oportunidades entre las 8 y 9 de la mañana, el intervalo de tiempo entre cada medición fue de tres minutos. Para el análisis, se consideró el promedio de las tres mediciones. Fueron utilizados tensiómetros aneroides con brazalete de velcro para adultos pequeños Riester exacta® y estetoscopios Riester Duplex®. Durante la medición, el estudiante estuvo sentado y con el brazo flexionado en una posición adecuada (apoyado sobre la mesa) y con el manguito inflable cubriendo dos tercios del largo y la circunferencia del brazo.

Para la extracción de las muestras sanguíneas, la venopunción fue realizada por un tecnólogo médico, con el estudiante en ayunas. La cantidad de sangre total recolectada fue de 5 mL en un tubo de polietileno tereftalato con activador coagulante y gel. Las muestras fueron trasladadas a un laboratorio privado en un periodo de una hora después de la toma de muestra; posterior a ello, fueron centrifugadas y separadas en alícuotas para ser procesadas el mismo día de la toma sanguínea.

Las pruebas de laboratorio fueron la determinación de los niveles de glucosa, el colesterol total y triglicéridos en suero (en mg/dL). Estos procedimientos fueron realizados con el método enzimático y con lecturas en espectrofotómetro. La determinación de colesterol HDL fue mediante el método colorimétrico sin precipitación. Para todas las pruebas, los reactivos usados fueron de la marca Wiener y el analizador automático bioquímico Wiener modelo CB 400i.

### Análisis estadístico

Se evaluó si la edad, presión arterial sistólica, presión arterial diastólica, talla, peso, perímetro de cintura, colesterol HDL, IMC, triglicéridos y glucosa en ayunas tuvieron distribución normal mediante la prueba de Kolmogorov-Smirnov. Se empleó la media y desviación estándar o mediana con rango intercuartílico (RIC) según el tipo de distribución de la variable cuantitativa. La diferencia de medias y medianas entre mujeres y varones se evaluó mediante la prueba t de Student y U de Mann-Whitney; respectivamente. Se presentan los componentes del SM de cada criterio diagnóstico mediante frecuencias y proporciones expresados en porcentajes, según sexo y toda la muestra. Además, se estimó la proporción de SM y sus intervalos de confianza al 95% (IC 95%).

El coeficiente Kappa de Cohen fue estimado para evaluar la concordancia entre los cinco criterios. Se consideró los siguientes niveles ^27^: leve (0,01 a 0,20), aceptable (0,21 a 0,40), moderado (0,41 a 0,60), sustancial (0,61 a 0,80) y casi perfecto (0,81 a 1,00). El porcentaje de acuerdo total entre los criterios fue calculado como la suma de acuerdos en resultados positivos y negativos entre el total de la muestra. La evaluación de la concordancia fue realizada para toda la muestra y según sexo. El análisis estadístico fue realizado en el programa libre Jamovi 2.2.5., y se consideró un valor de p<0,05 como estadísticamente significativo.

### Aspectos éticos

El estudio original fue aprobado por el Comité de Ética en Investigación de la Universidad Nacional de Cajamarca (código 009-2019). En dicha investigación, se solicitó mediante consentimiento informado la autorización para el uso futuro de los datos. Se analizó una base de datos que se encontraba anonimizada desde la fecha de término del análisis estadístico formal del estudio original (agosto 2021).

## RESULTADOS

### Características de la muestra

Fueron analizados los datos de 397 adolescentes, de los cuales, 196 fueron varones (49,4%). La mediana de la edad fue 14 años, la mitad tuvo de 13 a 16 años. Entre los varones, la mediana de la presión arterial sistólica, presión arterial diastólica, talla y peso fue mayor que en las mujeres. Por otro lado, en las mujeres, la mediana de colesterol HDL, IMC y triglicéridos fue mayor que en varones (Tabla 2).

**Tabla 2:** Características de los adolescentes enrolados en dos instituciones educativas de una zona altoandina del Perú.

Variable	Total (N = 397)		Mujeres ^b^ (n = 201)		Varones ^b^ (n = 196)		Valor de p ^a^
	Mediana	RIC	Mediana	RIC	Mediana	RIC	
Edad (años)	14	13 - 16	14	13 - 16	14	13 - 16	0,032
Presión sistólica media (mmHg)	100,0	90,0 - 103,3	93,3	90,0 - 102,0	100	93,3 - 107,0	<0,01
Presión diastólica media (mmHg)	63,3	60,0 - 70,0	60,0	60,0 - 70,0	66.7	60,0 - 70,0	<0,01
Talla (m)	1,52	1,47 - 1,58	1,49	1,46 - 1,54	1,57	1,50 - 1,62	<0,01
Peso (kg)	50,8	44,4 - 56,8	49,5	44,0 - 55,1	53,0	46,2 - 58,9	<0,01
Perímetro de la cintura (cm)	72,0	68,0 - 78,0	71,0	67,0 - 77,0	73,0	68,0 - 78,3	0,110
HDL (mg/dL)	38,0	33,0 - 43,0	38,0	34,0 - 44,0	36,0	30,0 - 42,0	<0,01
IMC (kg/m^2^)	21,5	19,7 - 23,8	21,9	20,1 - 24,0	21,2	19,2 - 23,5	0,028
Triglicéridos (mg/dL)	98,0	76,0 - 134,0	103,0	79,0 - 142,0	94,5	72,8 - 126,0	0,035
Glucosa (mg/dL)	80,0	74,0 - 86,0	80,0	73,0 - 85,0	81,0	74,0 - 86,0	0,506

a Valor de p correspondiente a la prueba U de Mann Whitney.

b Las mujeres fueron enroladas en el Colegio «Juan XXIII», los varones fueron enrolados en el colegio «San Ramón».

RIC: rango intercuartílico, IMC: índice de masa corporal, colesterol HDL: colesterol de lipoproteínas de alta densidad.

### Componentes del síndrome metabólico

En el componente de obesidad central, el criterio de Ferranti identificó a la mayor proporción de adolescentes (n = 80; 20,2%). Los criterios de la IDF y la AHA identificaron un caso con glucosa por encima del punto de corte. El colesterol HDL bajo fue el componente de SM más frecuente, con una proporción de 34,8% con el criterio de la OMS hasta 90,4% con el criterio de Ferranti. Este último criterio también identificó la mayor proporción de hipertrigliceridemia (48,9%). Los criterios de la ATP-III, de Ferranti y de la AHA identificaron, cada uno, 25 adolescentes hipertensos (Tabla 3).

**Tabla 3:** Frecuencia de los componentes de síndrome metabólico según cinco criterios diagnósticos.

Criterio ^a^	Sexo ^b^		Total
	Varones (n = 196)	Mujeres (n = 201)	
	n (%)	n (%)	n (%)
ATP - III			
Síndrome metabólico	11 (5,6)	5 (2,5)	16 (4,0)
Obesidad central	5 (2,6)	7 (3,5)	12 (3,0)
Hiperglicemia	0 (0)	0	0
Hipertrigliceridemia	68 (34,7)	89 (44,3)	157 (39,5)
HDL bajo	135 (68,9)	123 (61,2)	258 (65,0)
Hipertensión arterial	19 (9,7)	6 (3,0)	25 (6,3)
De Ferranti			
Síndrome metabólico	39 (19,9)	29 (14,4)	68 (17,1)
Obesidad central	43 (21,9)	37 (18,4)	80 (20,2)
Hiperglicemia	0	0	0
Hipertrigliceridemia	86 (43,9)	108 (53,7)	194 (48,9)
HDL bajo	181 (92,3)	178 (88,6)	359 (90,4)
Hipertensión arterial	19 (9,7)	6 (3,0)	25 (6,3)
IDF			
Síndrome metabólico	5 (2,6)	7 (3,5)	12 (3,0)
Obesidad central	8 (4,1)	20 (10,0)	28 (7,1)
Hiperglicemia	0	1 (0,5)	1 (0,3)
Hipertrigliceridemia	31 (15,8)	41 (20,4)	72 (18,1)
HDL bajo	125 (63,8)	127 (63,2)	252 (63,5)
Hipertensión arterial	0	0	0
AHA			
Síndrome metabólico	9 (4,6)	6 (3,0)	15 (3,8)
Obesidad central	5 (2,6)	7 (3,5)	12 (3,0)
Hiperglicemia	0	1 (0,5)	1 (0,3)
Hipertrigliceridemia	68 (34,7)	89 (44,3)	157 (39,5)
HDL bajo	89 (45,4)	88 (43,8)	177 (44,6)
Hipertensión arterial	19 (9,7)	6 (3,0)	25 (6,3)
OMS			
Síndrome metabólico	11 (5,6)	3 (1,5)	14 (3,5)
Hiperglicemia	0	0	0
Hipertrigliceridemia	42 (21,4)	55 (27,4)	97 (24,4)
HDL bajo	85 (43,4)	53 (26,4)	138 (34,8)
Hipertensión arterial	0	0	0
IMC > P95	20 (10,2)	10 (5,0)	30 (7,6)

HDL: colesterol de lipoproteínas de alta densidad, HTA: hipertensión arterial, IMC: índice de masa corporal, P95: percentil 95, ATP-III: *Third Adult Treatment Panel,* IDF: *International Diabetes Federation*, AHA: *American Heart Association,* OMS: Organización Mundial de la Salud.

a Los puntos de corte para cada componente de síndrome metabólico en adolescentes han sido definidos en la tabla 1.

b Las mujeres fueron enroladas en el Colegio «Juan XXIII», los varones fueron enrolados en el colegio «San Ramón».

### Frecuencia de síndrome metabólico

El criterio de Ferranti clasificó como SM al 17,1% de adolescentes (IC 95%: 13,4 a 20,8), el criterio ATP-III identificó al 4,0% (IC 95%: 2,1 a 6,0), el criterio AHA al 3,8% (IC 95%: 1,9 a 5,7), el criterio de la OMS al 3,5% (IC 95%: 1,7 a 5,3), y el criterio de la IDF fue el que menos casos identificó (3,0%; IC 95%: 1,3 a 4,7). Los criterios de Ferranti, ATP-III, AHA y OMS identificaron a una mayor proporción de casos de SM entre varones (Tabla 3).

### Concordancia de criterios

El mayor porcentaje de acuerdo total estuvo entre los criterios de la AHA y de la ATP-III (99,2%). El criterio de la IDF tuvo porcentajes de acuerdo de 97,0% y 97,7% con la AHA y la OMS; respectivamente. Las proporciones de acuerdo entre los criterios difieren entre varones y mujeres (Tabla 4).

**Tabla 4:** Número de casos concordantes y porcentaje de acuerdo entre cinco criterios diagnósticos de síndrome metabólico.

Grupo	ATP III				De Ferranti				IDF				AHA			
	+	-	Total	%	+	-	Total	%	+	-	Total	%	+	-	Total	%
Total																
De Ferranti	17	329	346	87,2												
IDF	7	375	382	96,2	11	328	339	85,4								
AHA	15	379	394	99,2	16	329	345	86,9	8	377	385	97,0				
OMS	8	375	383	96,5	13	329	342	86,1	8	380	388	97,7	8	376	384	96,7
Varones																
De Ferranti	11	157	168	85,7												
IDF	4	184	188	95,9	5	157	162	82,7								
AHA	9	185	194	99,0	9	157	166	84,7	4	186	190	96,9				
OMS	6	181	187	95,4	10	157	167	85,2	5	186	191	97,4	6	183	189	96,4
Mujeres																
De Ferranti	6	172	178	88,6												
IDF	3	191	194	96,5	6	171	177	88,1								
AHA	6	194	200	99,5	7	172	179	89,1	4	191	195	97,0				
OMS	2	194	196	97,5	3	172	175	87,1	3	194	197	98,0	2	193	195	97,0

+: acuerdo en casos positivos, -: acuerdo en casos negativos, %: porcentaje de acuerdo total.

ATP-III: *Third Adult Treatment Panel*, IDF: *International Diabetes Federation*, AHA: *American Heart Association*, OMS: Organizacion Mundial de la Salud.

Se encontró un acuerdo «casi perfecto» entre los criterios de la AHA y de la ATP-III (k = 0,905), este nivel de concordancia se observó en varones (k = 0,895) y en mujeres (k = 0,921). Entre los criterios de la OMS y de la IDF (k = 0,628) se encontró un acuerdo «sustancial», sin embargo, dicho nivel de acuerdo no se mantuvo entre mujeres (k = 0,592). Se encontró un nivel de acuerdo «moderado» entre el criterio de la IDF con el de ATP-III y de la AHA, y del criterio de la OMS con el de la ATP-III y la AHA. El criterio de Ferranti tuvo un nivel de acuerdo «aceptable» con los demás criterios (Figura 1).

**Figura 1:** Estimación de la concordancia entre cinco criterios diagnósticos de síndrome metabólico entre escolares de dos instituciones educativas públicas de un distrito altoandino (Cajamarca, Perú).

Los cinco criterios empleados coincidieron en clasificar como SM a 6 adolescentes (1,5%), cuatro criterios coincidieron en 3 (0,8%), tres criterios en 9 (2,3%) y dos criterios en 6 (1,5%). Finalmente, 45 (11,3%) adolescentes fueron identificados como SM por alguno de los criterios empleados.

## DISCUSIÓN

Entre adolescentes que residen en una zona altoandina del Perú, los criterios de la AHA y de la ATP-III tuvieron una concordancia «casi perfecta» para identificar SM, por otro lado, el criterio de Ferranti tuvo los menores niveles de concordancia con respecto a los criterios de la IDF, de la AHA, la OMS y la ATP-III. El coeficiente de concordancia estimado entre los criterios de la AHA y la ATP-III fue superior a lo encontrado en un estudio realizado en 1004 adolescentes europeos de 12,5 a 17 años. En dicho estudio, los criterios de la AHA y la ATP-III alcanzaron un kappa entre 0,73 a 0,80; sin embargo, al igual que nuestro estudio, dicho coeficiente fue el mayor entre aquellos generados por la comparación entre cinco criterios diferentes (ATP-III, AHA, OMS, IDF y los de Jolliffe & Janssen) ^14^. 

Este nivel de concordancia (entre AHA y ATP-III) podría explicarse porque ambos criterios solo se diferencian en la definición de un componente. El criterio de la ATP-III modificado por Cook considera colesterol HDL bajo cuando sus valores son inferiores a 40 mg/dL, mientras que la AHA usa valores iguales o por debajo del percentil 10 para raza y sexo. Debido a la ausencia de los valores referenciales para adolescentes peruanos o residentes en zonas altoandinas, se usaron los valores de los percentiles definidos por una encuesta nacional de salud y nutrición de los Estados Unidos de Norteamérica ^28^, a partir de la cual, Cook definió el valor de 40 mg/dL para el colesterol HDL.

Se encontró una concordancia «sustancial» entre los criterios de la OMS y de la IDF, solo un estudio ha reportado la concordancia entre dichos criterios. En adolescentes europeos se estimó un coeficiente kappa entre 0,29 y 0,38 ^14^, que, según los cortes aplicados en nuestro estudio, corresponde a una concordancia «aceptable». El criterio de la OMS es diferente a los otros: i) utiliza el IMC en lugar de la circunferencia de cintura; ii) define hipertensión a los valores igual o superior al percentil 95 de presión arterial; y iii) utiliza el corte más restrictivo para definir colesterol HDL bajo (< 35 mg/dL). A pesar de estas diferencias, se encontró una concordancia con el criterio de la IDF superior a la previamente reportada. Entre las explicaciones para ese resultado están que ambos criterios no identificaron casos de hipertensión arterial, y son los que plantean los cortes más altos y próximos entre sí para definir hipertrigliceridemia.

La concordancia entre los criterios de la ATP-III y de la IDF ha sido la más estudiada entre adolescentes de diversas nacionalidades con concordancias muy diversas. En Latinoamérica, entre 241 adolescentes de 10 a 14 años del estado de Paraná en Brasil, el acuerdo total entre dichos criterios fue 97,5%, y el coeficiente kappa fue 0,95 ^12^. Otro estudio realizado en 1200 adolescentes de 12 a 17 años del estado de Santa Cruz do Sul en Brasil estimó un kappa de 0,53 ^15^. En 851 adolescentes entre 10 a 18 años de la ciudad de Medellín en Colombia se estimó un kappa de 0,39 ^10^; mientras que en 916 niños y adolescentes de 9 a 18 años de la ciudad de Mérida en Venezuela se estimó un kappa de 0,28 ^29^. Entre los estudios en Europa, uno realizado en 379 adolescentes españoles de 12 a 16,9 años encontró un kappa de 0,82 ^30^. En otro estudio realizado en adolescentes de Italia de 10 a 16 años el kappa fue 0,71 ^31^, mientras que en 1004 adolescentes residentes en 10 ciudades europeas se estimó un kappa de 0,47 ^14^. 

Los diferentes coeficientes de concordancia entre los criterios ATP-III e IDF se explicarían porque si bien usan los mismos componentes, el criterio IDF requiere obligatoriamente la presencia de obesidad central, componente que, a su vez, es determinado de manera diferente al ATP-III. El criterio IDF aplica un corte determinado por el percentil 90 para los menores de 16 años, mientras que para aquellos de 16 años o más usa puntos de corte definidos en 94 y 80 cm para varones y mujeres, respectivamente. Otras diferencias son los puntos de corte para definir hiperglicemia e hipertrigliceridemia, el corte para glicemia en IDF es menor comparado con ATP-III; mientras que el corte para la concentración de triglicéridos es mayor en ATP-III. Finalmente, IDF usa puntos de corte fijos (≥130/85 mmHg) para definir la hipertensión arterial, mientras que ATP-III usa percentiles. A partir de estas diferencias, el criterio ATP-III clasifica una mayor proporción de adolescentes como SM respecto al IDF, esto ha sido observado en Brasil ^12^, España ^30^, Colombia ^10^, en diversos países europeos ^14^, y en nuestro estudio.

El criterio de Ferranti identificó el SM en una mayor proporción de adolescentes (17,1%), es decir, en promedio identificó tres a cuatro veces más casos respecto a los demás criterios. Este hallazgo ha sido reportado en otros estudios, por ejemplo, en Brasil, el criterio de Ferranti identificó como SM al 17,4% de adolescentes, mayor a lo identificado por el criterio de ATP-III modificado por Cook (3,3%) e IDF (1,7%) ^12^. En Colombia, el criterio de Ferranti encontró un 11,4% de SM, igualmente, superior a los criterios de ATP-III modificados por Cook (3,8%) e IDF (0,9%) ^10^. Este hallazgo se debe a los puntos de corte menos restrictivos de los criterios de Ferranti para definir obesidad central, hipertrigliceridemia y niveles de colesterol bajo. 

Se encontró que el colesterol HDL bajo fue el componente más frecuentemente identificado por todos los criterios. El criterio de Ferranti definió este componente en el 90,4% de adolescentes, seguido de los criterios de la ATP-III modificados por Cook (65%) y el de IDF (63,5%). El segundo componente más frecuente fue la hipertrigliceridemia, de igual manera el criterio de Ferranti identificó con esta condición al 48,9% de adolescentes, seguido de los criterios de la ATP-III y la AHA. Este patrón es similar a lo encontrado en adolescentes de Colombia ^10^, pero difiere de lo encontrado en otras poblaciones de adolescentes. Por ejemplo, en adolescentes españoles el componente más frecuente fue la obesidad abdominal ^13^^,^^30^, al igual que en adolescentes mexicoamericanos de los EE. UU. ^32^; mientras que en Brasil, el componente más frecuente fue la hipertensión arterial ^12^^,^^15^.

Diversos estudios muestran un perfil lipídico particular en pobladores adolescentes y adultos de los Andes, caracterizado por elevados niveles de triglicéridos séricos, bajos niveles de colesterol HDL e hipercolesterolemia ^3^^,^^33^, dicho perfil también ha sido encontrado en pobladores adultos tibetanos (3660 m s. n. m.) ^34^, mientras que en China en adultos que residen a más de 2000 m s. n. m., la prevalencia de hipertrigliceridemia o colesterol HDL bajo fue del 9,1% ^17^. Se reporta que las dislipidemias pueden ser un problema frecuente en adolescentes. La elevada frecuencia de dislipidemias encontrada en adolescentes que residen a casi 3000 m s. n. m. podría ser explicada por factores genéticos ^35^, y la influencia de las adaptaciones metabólicas y hormonales a la altura; sin embargo, esta hipótesis requiere de estudios específicos. 

Solo los criterios de la IDF y la AHA identificaron un caso de glicemia elevada, ambos criterios tienen como punto corte 100 mg/dL. La baja frecuencia de este componente ha sido previamente reportada, con un corte de 110 mg/dL en adolescentes de la ciudad de Cajamarca ^3^ y también en otros estudios de concordancia de criterios de SM en Colombia ^10^, Brasil ^11^^,^^15^ y España ^13^. Este hallazgo es consistente con las adaptaciones de la respuesta celular a la hipoxia crónica a elevadas alturas ^17^^,^^36^, y por la baja frecuencia de obesidad abdominal y el consecuente bajo riesgo de resistencia a la insulina. 

Lo descrito en este análisis de concordancia es una manifestación del complejo diagnóstico del SM en adolescentes. Estos resultados tienen implicancias en futuras investigaciones en adolescentes que residen en altura, pues se observa lo crucial que es la selección del criterio diagnóstico y cómo esta decisión puede afectar las estimaciones de frecuencia del SM. En general, el criterio de Ferranti clasifica como SM a una mayor proporción de adolescentes, este hallazgo no implica un mejor rendimiento diagnóstico, de hecho, es el criterio que menor concordancia guarda con los demás. De manera secundaria, el presente análisis identifica como potencial problema de salud pública a la elevada frecuencia de hipertrigliceridemia y colesterol HDL bajo. Por ello, el diagnóstico del SM en adolescentes requiere criterios que respeten la naturaleza continua de sus componentes y la construcción de puntajes que definan el riesgo cardiometabólico en base a datos poblacionales y representativos en diferentes pisos altitudinales. Definir adecuadamente la presencia de cada componente permitirá mejorar la identificación del SM, y establecer estrategias de intervención y prevención en adolescentes con o sin SM.

El estudio presenta limitaciones. No se disponían de los valores de percentiles para la presión arterial sistólica y diastólica, ni para colesterol HDL, por lo que fueron utilizados datos disponibles de la NHANES III realizada en los EE. UU. Al ser un estudio de fuentes secundarias, no se realizó un cálculo *a priori* del tamaño muestral para estimar coeficientes de concordancia. El criterio de la OMS evalúa la homeostasis glicémica mediante los niveles de insulina, la intolerancia a la glucosa y glucosa en ayunas, en este análisis solo se empleó la última. Por último, varios criterios usan puntos de corte arbitrarios para definir la presencia de un componente, especialmente para hiperglicemia e hipertrigliceridemia; con los cambios en los estilos de vida y factores de riesgo de las poblaciones, así como de los procedimientos laboratoriales, dichos cortes pueden variar y requieren reevaluarse. Asimismo, el presente estudio debe interpretarse con cautela. Primero, estudiamos un solo distrito ubicado en los Andes del Perú, por lo que, en vista de los diversos pisos altitudinales, estilos de vida y etnias, los resultados no son representativos para los adolescentes que viven en los andes peruanos. Segundo, nuestros resultados no evalúan ni concluyen qué criterio tiene mayor rendimiento diagnóstico para SM, para ello deben realizarse estudios que evalúen la validez de criterio respecto a un diagnóstico de referencia. La complejidad de ello radica en que a la fecha no existe un consenso para el diagnóstico de SM en adolescentes. 

En conclusión, en adolescentes escolares residentes de una zona altoandina en la ciudad de Cajamarca, Perú, los cinco criterios empleados concuerdan en menos del 2% para identificar al mismo grupo como SM, lo que genera estimaciones de prevalencias diferentes. Los criterios de la AHA y ATP-III modificados por Cook tuvieron una concordancia «casi perfecta», este nivel de concordancia se mantuvo en ambos sexos. El criterio de Ferranti identificó SM en 17,1% de adolescentes, este criterio identificó tres a cuatro veces más SM que los demás.
